# Blood pressure telemonitoring and the incidence of cardiovascular events: a records based, matched patient analysis

**DOI:** 10.1093/ehjdh/ztag069

**Published:** 2026-05-26

**Authors:** Janet Hanley, Mary Paterson, Richard Parker, Alice Pearsons, Roz Pollock, Lis Neubeck, Iain Atherton, Brian McKinstry

**Affiliations:** Edinburgh Napier University, School of Health and Social Science, Sighthill Campus, Edinburgh EH11 4BN, UK; Edinburgh Napier University, School of Health and Social Science, Sighthill Campus, Edinburgh EH11 4BN, UK; University of Edinburgh, Usher Institute, Edinburgh, UK; Edinburgh Napier University, School of Health and Social Science, Sighthill Campus, Edinburgh EH11 4BN, UK; University of Edinburgh, Usher Institute, Edinburgh, UK; Edinburgh Napier University, School of Health and Social Science, Sighthill Campus, Edinburgh EH11 4BN, UK; Edinburgh Napier University, School of Health and Social Science, Sighthill Campus, Edinburgh EH11 4BN, UK; University of Edinburgh, Usher Institute, Edinburgh, UK

**Keywords:** Hypertension, blood pressure, telemonitoring, observational study

## Abstract

**Aims:**

It is well established from trials that blood pressure (BP) telemonitoring leads to improved BP control. However, there is little data available on the impact of BP telemonitoring on the incidence of cardiovascular events when it is used as the routine mode of long-term BP monitoring. The objective of this study was to assess the impact of BP telemonitoring on cardiovascular outcomes.

**Methods and results:**

Records were analysed for 454 180 adults with hypertension who had a prescription for a first-line anti-hypertensive drug at any time from 1 March 2019 to 28 February 2021. Follow-up was until 1st March 2022. Women pregnant during that time were excluded. The primary outcome was emergency hospital admission or mortality for Acute Coronary Syndrome (ACS), Stroke, or uncontrolled Heart Failure (HF) within 12 months. Outcomes were compared between people who had used BP telemonitoring for at least 1 year and a group who had never used it, matched for age, sex, ethnicity, social deprivation, number of anti-hypertensive drugs, diabetes, and having a BP assessment in the same year. Ninety percent of the cohort had been diagnosed with hypertension before March 2019. The onset of the COVID-19 pandemic in March 2020 was associated with a rapid increase in the uptake of BP telemonitoring but a reduction in the number of new diagnoses of hypertension. Those who used telemonitoring were significantly younger, less likely to have diabetes, and took less antihypertensive medication. For those who used telemonitoring for over 1 year, a mean reduction in systolic BP was seen by 3 months which was maintained for at least the remainder of the year.

**Conclusion:**

In the matched cohort analysis, people who used telemonitoring were less likely to be admitted to hospital with or die from ACS, stroke, or uncontrolled HF telemonitoring [adjusted OR 0.665 (0.501 to 0.884), *P* = 0.0049] than those who were not using telemonitoring.

## Graphical Abstract

**Graphical Abstract:**
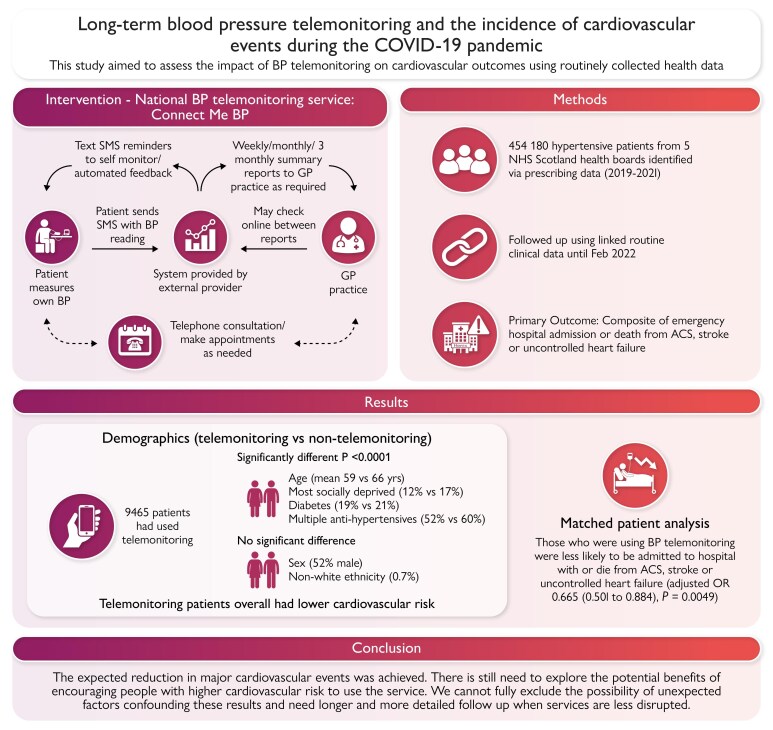

## Introduction

Hypertension is a major global health problem, affecting an estimated 1.28 billion adults worldwide.^1^ Uncontrolled hypertension is a leading cause of cardiovascular diseases, including acute coronary syndrome (ACS), stroke, and heart failure (HF), contributing significantly to the global burden of morbidity and mortality.^2^ The World Health Organization (WHO) reports that hypertension is responsible for approximately 7.5 million deaths annually, about 12.8% of all deaths.^3^ However, reduction in blood pressure (BP) using lifestyle measures or medication reduces the risk, with that risk reducing proportionately with levels of systolic BP down to and below the 130–140 mmHg recommended in many guidelines.^4–7^ Uncontrolled hypertension is largely asymptomatic and so regular BP monitoring is required following diagnosis. Long-term BP telemonitoring, where an individual measures their BP at home at regular intervals and shares it electronically with their healthcare provider, has several advantages over traditional surgery-based monitoring. These include better BP control than either surgery-based monitoring or home BP monitoring alone (even when supported by a smartphone app) and convenience.^8,9^ The mechanisms underpinning improved BP control include increased trust in the measurement and empowerment of patients to proactively approach their healthcare provider; reducing delays in optimizing anti-hypertensive therapy^10,11^ . The hypothesis is therefore that use of BP telemonitoring by people with hypertension as part of routine health care will result in a reduction in the incidence of major cardiovascular events compared with a group of similar patients who do not use it.

**Figure 1:**
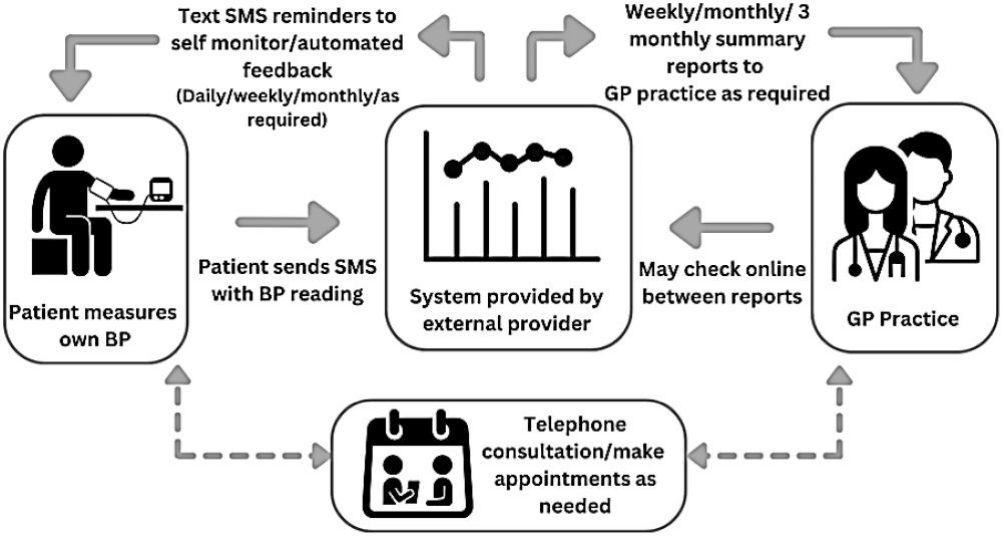
Connect Me BP blood pressure telemonitoring system.

In Scotland (population 5.48 million), 17% of those over 16 years have diagnosed hypertension and a further 12% thought to have undiagnosed hypertension.^12^ Health care, including all prescribed medication, is provided free at the point of care, paid for through taxation. Hypertension is largely managed in primary care by practice nurses and pharmacists supported by family doctors. Prior to the start of the COVID-19 pandemic in March 2020, around 13% of all consultations were for BP management. Scotland, the site of this study, has also established a BP telemonitoring service (Connect Me BP) provided through family medical practices as an adjunct to standard care. *Figure 1* shows a flow chart of the service, which can be used flexibly, with the clinician able to dynamically adjust both the frequency of readings and reporting. This had been set up in one region in 2015 (then called Scale up BP) as a pilot service and an implementation study showed that people with established hypertension who used telemonitoring achieved similar reductions in BP to those achieved in RCTs.^13^ At the time, there were too few users to determine the effect on cardiovascular outcomes, including stroke, ACS, and uncontrolled HF. The service was extended to the rest of Scotland as Connect Me BP in 2018. The purpose of this study was to update the earlier study of the implementation of this service^13^ and go beyond the focus on change in BP demonstrated in that study to compare clinical cardiovascular outcomes for those using BP telemonitoring with a matched group who did not use the service. Patients in this standard care group would normally have their blood pressure measured in the surgery by nurses from their family doctor's practice.

However, it should be noted that, given the service start date, evaluation of cardiovascular outcomes from this service includes the period of the COVID-19 pandemic, declared in March 2020, which caused changes to service provision and patient behaviour.^14^ Reduced rates of hypertension diagnosis and prescribing were reported globally, and in Scotland, routine face-to-face monitoring for many conditions, such as hypertension, was paused.^15,16^ Consequently, the remote monitoring capacity of the Connect Me BP service became especially important, which drove rapid expansion of the service. Even where face-to-face care was available, patients were unwilling to attend as they were aware that hypertension had been associated with increased susceptibility to COVID-19 infection and adverse outcomes, including severe disease progression and mortality.^17^ We are aware that during the COVID-19 pandemic, some patients in the standard care group were loaned a BP monitor by the practice for a week in order to take their BP daily at home for 1 week and return the results on paper, but we do not know how widespread this practice was.

There was also a reported drop in non-COVID-19 emergency presentations at hospital,^18^ although it is not clear if this affected emergency hospital admission for ACS, stroke or uncontrolled HF.

## Methods

Records were analysed for patients from five Scottish Health Boards (published adult population 2 362 494) who were over the age of 16 and had a prescription dispensed for at least one of the first-line anti-hypertensive medications recommended in guidance issued by the National Institute of Clinical Excellence (NICE).^19^ Patients were included if they had a prescription dispensed between 1 March 2019 (one year before the first cases of COVID-19 were identified in Scotland) and 28 February 2021. Follow-up data were collected until 28 February 2022, so each patient contributed at least 1 year of data. Women who had been pregnant during that period were excluded using the SMR04 Scottish maternity record. The drug classes used to identify patients were angiotensin-converting enzyme inhibitors (ACE), angiotensin receptor blockers (ARB), and calcium channel blockers (CCB). The drug list from the British National Formulary is shown in the Supplementary material. It was estimated that this population would yield an estimated 404 200 people with hypertension based on the prevalence of 17% of the adult population with identified and treated hypertension from the Scottish Health Survey 2019.^12^ To check the coverage of this sampling technique, diagnostic and prescribing data were obtained from a sub-sample of 38 family practices with a look-back period of at least 5 years for a diagnosis of hypertension (to 2014).

Family practices were recruited through the NHS Research Scotland Primary Care network. Practices that provided data gave written permission and the data were extracted and transferred securely to Public Health Scotland (PHS) for linkage and pseudonymization. To maintain anonymity, these data were not directly linked to the large dataset based on prescribing data and were analysed separately. PHS linked and pseudonymized the data and transferred it to the secure National Safe Haven analysis platform for access by the research team. Data were linked deterministically based on the Scottish Community Health Index (CHI) number, which is attached to all health records. Data included:

- Demographic data, including date of birth, sex, and deprivation status as measured by the Scottish Index of Multiple Deprivation (SIMD) 2020^20^ from the CHI database
- BP telemonitoring data from the Connect Me BP service
- Outcome data (cardiovascular events and mortality) plus all-cause hospital admission and outpatient attendance records from Scottish Morbidity Records and National Records of Scotland
- Covid testing and vaccination records from PHS
- Clinical data from a sub-sample of 38 family practices.

Patients were included in the telemonitoring cohort if they had submitted more than one reading to the system. Patients who had submitted only one reading were excluded from both telemonitoring and non-telemonitoring cohorts, as this may have only been a demonstration reading taken in the surgery, so they had been exposed to telemonitoring but had not used it. Patient records were excluded if contradictory dates suggested a linkage error or missing data. BP readings were excluded if one component of the reading was missing or the systolic pressure was lower or within 10 mmHg of the diastolic reading. Systolic BP readings of < 55 mmHg or >350 mmHg were reclassified as missing (there were no readings near those limits; most out-of-range readings were due to both the systolic and diastolic readings having been entered without a delimiter between them). Less than 1% of readings were reclassified. One-way analysis of variance (ANOVA) was used to examine mean systolic BP by month from the commencement of telemonitoring for people who started telemonitoring within the timeframe of this study and had 1 year of measurements.

The primary cardiovascular outcome of interest was a composite of emergency admission to hospital or death due to ACS, stroke, or transient ischaemic attack (TIA) or uncontrolled HF. This has been used in previous major studies.^6^ Outcome events were identified from ICD-10 codes recorded in the Scottish Morbidity Record (SMR) data. The ICD-10 coding used was checked with PHS, who maintain the Scottish Morbidity Record (SMR) and is available in the supplementary data. A secondary outcome was the change in telemonitored BP. The purpose of this was to determine if the early reduction in BP with the introduction of telemonitoring in people whose hypertension had been treated for more than 6 months, seen in RCTs, was also seen in this population.

For the comparative analysis, telemonitoring patients were matched to controls according to the following variables: age, sex, SIMD quintile, on diabetes medication or diabetes admission/clinic (yes/no), ethnicity status (White, other, missing), number of different classes of anti-hypertension medications in the first 6 months, and index date (before March 2020, March 2020 or later). The index date referred to the first BP monitoring date for telemonitoring patients or the first prescribing date of medications for control patients, both of which were markers that an assessment of BP control had been carried out. Only patients with a full year of telemonitoring data were included in this analysis. We performed exact matching of telemonitoring patients to control patients using a variable ratio exact matching strategy. Matched sets (strata) were defined by unique combinations of the matching variables, and all individuals sharing an identical profile across these variables were assigned to the same matched stratum. This approach yielded variable-sized matched sets (i.e. 1:K matching), where each telemonitoring patient could be matched to one or more controls, and each control patient could be matched to one or more telemonitoring patients. Duplicates with respect to identical patient identifiers were removed such that each patient appeared only once in the matched dataset as a whole, and patients did not appear in more than one matched set (i.e. matched sets were non-overlapping). However, each matched set could contain multiple telemonitoring or control patients if they have the same covariate profiles. Matching was performed using cumulative sampling, as controls were selected based on matching variables rather than on being at risk at a specific exposure time. We then used the matched data (merged with the original dataset) to analyse the association between telemonitoring and outcomes using conditional logistic regression, conditioning on the matched strata. This method appropriately accounts for the dependence introduced by the matching process and implicitly adjusts for all matching variables by ensuring that comparisons are made only within strata of identical covariate profiles.

SAS software version 9.4 was used to perform the conditional logistic regression modelling. Stratified analysis was also performed, stratifying by index date.

## Results

**Figure 2:**
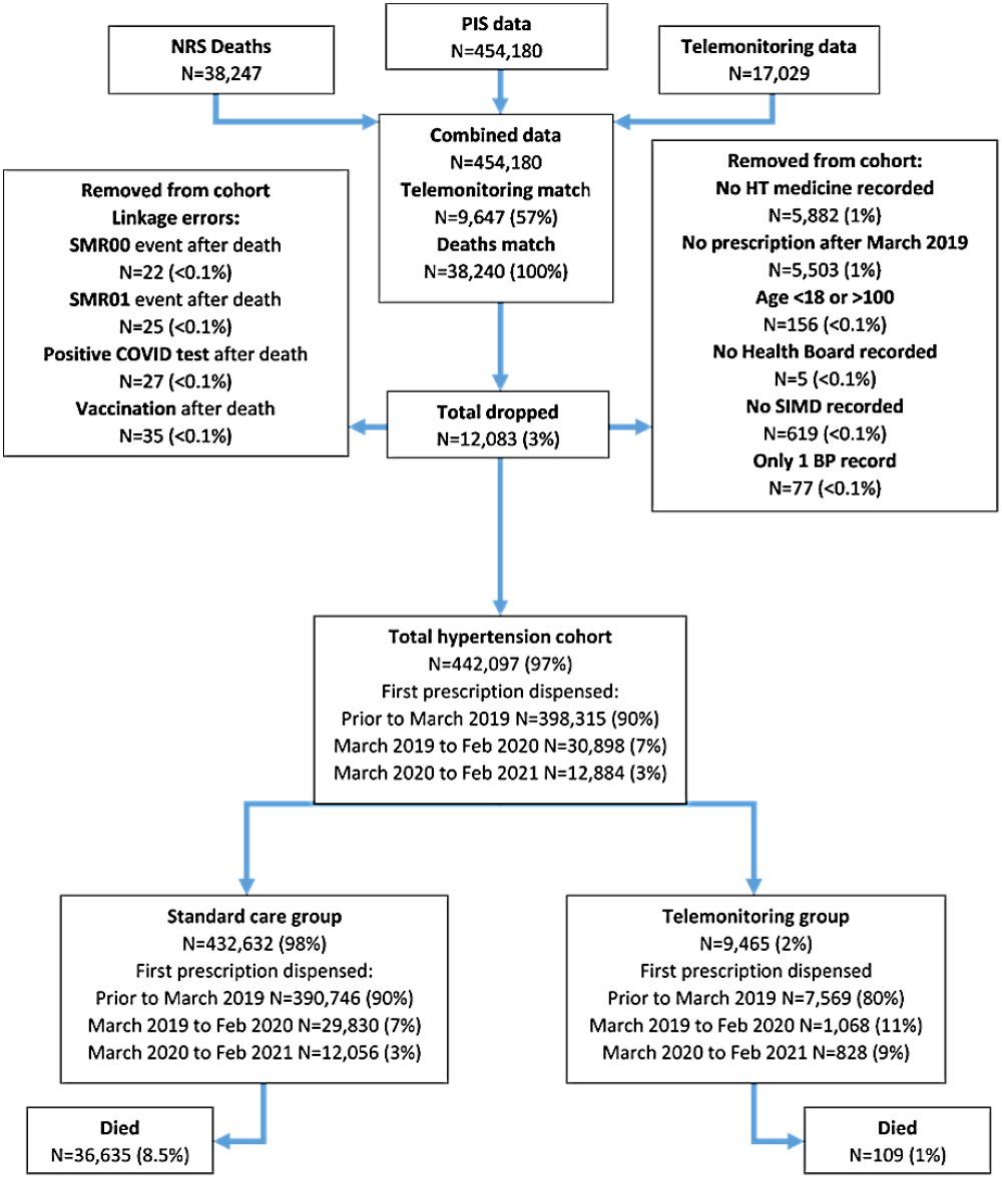
Data flow in the main cohort.

**Figure 3:**
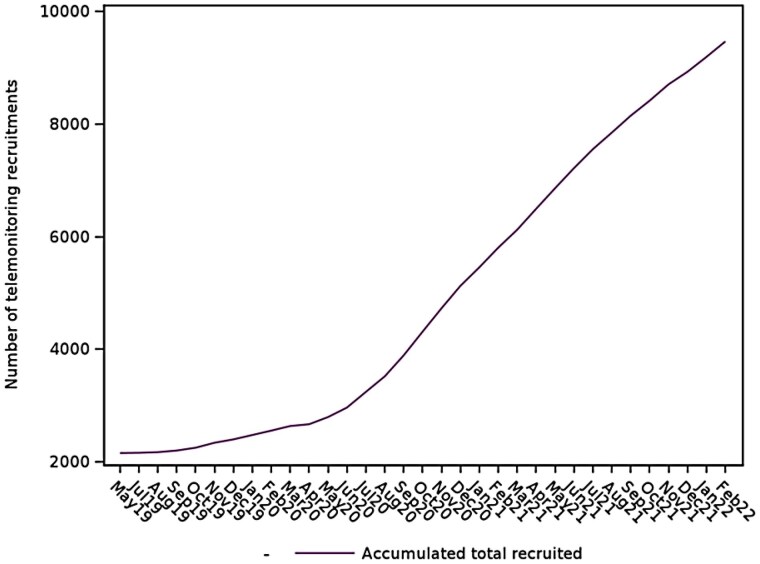
Number of patients starting to use telemonitoring by date.

Reporting was guided by the Strengthening the Reporting of Observational Studies in Epidemiology (STROBE) standards.^21^ The data flow for this cohort are shown in *Figure 2*. The population characteristics are shown in *Table 1*. Ninety percent of those identified had been diagnosed with hypertension prior to March 2019. The number of people diagnosed with hypertension during the first year of the COVID-19 pandemic was less than half of that of the previous year. There were significant demographic differences between the group of patients who were telemonitoring and those who were not, except for ethnicity and sex, which were similar between groups. As *Figure 3* shows, the number of users using telemonitoring increased from 2040 to 9465 by February 2022. The mean number of BP readings submitted by these users was 60 (SD 96.0) with a median of 33 (interquartile range 21–66).

**Table 1:** Characteristics of the cohort

		Standard care *n* = 432632	BP telemonitoring *n* = 9465	*P*-value
		*n* (%)	*n* (%)	
**Diagnosed**	*Pre March 2019*	39 0746 (90%)	7569 (80%)	<0.0001
	*March 2019–Feb 2020*	29 830 (7%)	1068 (11%)	
	*March 2020–Feb 2021*	12 056 (3%)	828 (9%)	
**Mean age (SD)**	Range (both groups) < 25 – > 90 years	66 years (12.6)	59 yrs (10.8)	<0.0001
**Sex**	Male	225 739 (52%)	5017 (53%)	0.113
**Scottish Index of Multiple Deprivation (2020)**	*1 (most deprived)*	72 674 (17%)	1148 (12%)	<0.0001
	*2*	99 275 (23%)	2210 (23%)	
	*3*	83 528 (19%)	1810 (19%)	
	*4*	86 480 (20%)	1895 (20%)	
	*5 (least deprived)*	90 675 (21%)	2402 (25%)	
**Ethnic Group (missing in 33% of records)**	White	258 699 (99.3%)	5133 (99.2%)	0.93
	Other	1926 (0.7%)	43 (0.8%)	
**Number of classes of antihypertensive drugs in 6 months**	1	174 662 (40%)	4526 (48%)	<0.0001
	*2*	165 165 (38%)	3253 (34%)	
	*3*	68 993 (16%)	1275 (14%)	
	*4 or more*	23 812 (6%)	411 (4%)	
**Classes of anti-hypertensive drugs**	*Angiotensin-converting enzyme inhibitors (ACE)*	240 415 (55.6%)	3731(60.9%)	
	*Angiotensin receptor blockers (ARB)*	103 556 (23.9%)	1426 (23.3%)	
	*Calcium channel blockers (CCB)*	211 949 (49.0%)	2960 (48.3%)	
	*Diuretic*	111 916 (31%)	1136 (19%)	
	*Beta blocker*	120 784 (28%)	1078 (18%)	
	*Other*	23 333 (5%)	376 (6%)	
**Diabetes**		89 514 (21%)	1765 (19%)	<0.0001
**Died**	All causes	36635 (8%)	109 (1%)	<0.0001

### Change in BP on commencement of telemonitoring

Data on the change in systolic BP were analysed for people who started telemonitoring during the period of study and had at least 1 year of potential use of the service. Mean systolic BP reduced from 141 mmHg (SD 14.6) in the first month of using the service to 133 mmHg (SD 12.0) after 3 months and this drop was sustained (repeated measures ANOVA *F* = 24.52, *P* < 0.0001). A table showing mean BP by month is available in the Supplementary material.

### Hospital admissions and mortality

**Figure 4:**
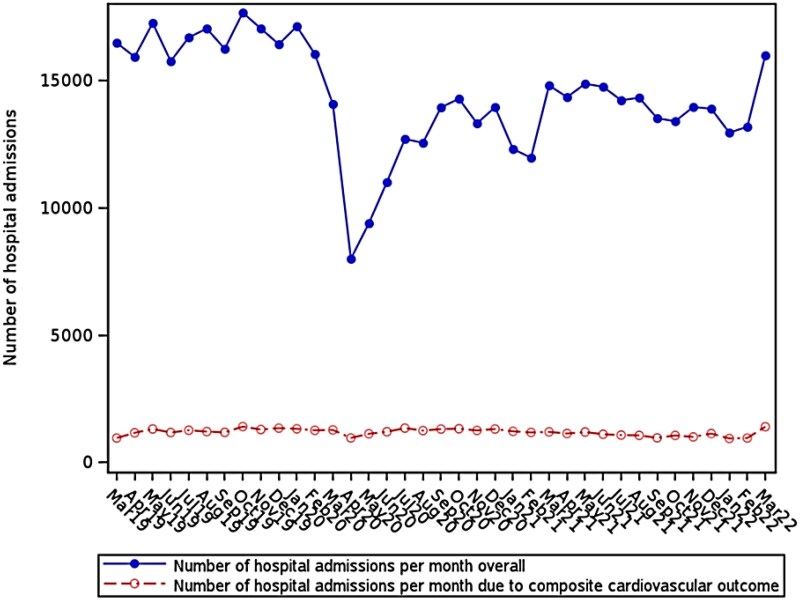
Hospital admissions (SMR01) and composite cardiovascular outcomes.

Over the 3-year period, 69% of the cohort attended hospital outpatient departments with the median number of visits being 4 (interquartile range 2–8). Also, 45% of the cohort had at least one inpatient admission to the hospital, with 55% of the admissions being emergencies. However, these were not evenly distributed over time (*Figure 4*), with a dramatic fall in hospital admissions at the start of the COVID-19 pandemic. However, as the graph shows, any effect of the pandemic on emergency hospital admissions for ACS, stroke, or uncontrolled HF was limited and very short-term. Four percent of patients (7950) had COVID-19-related hospital admissions.

**Figure 5:**
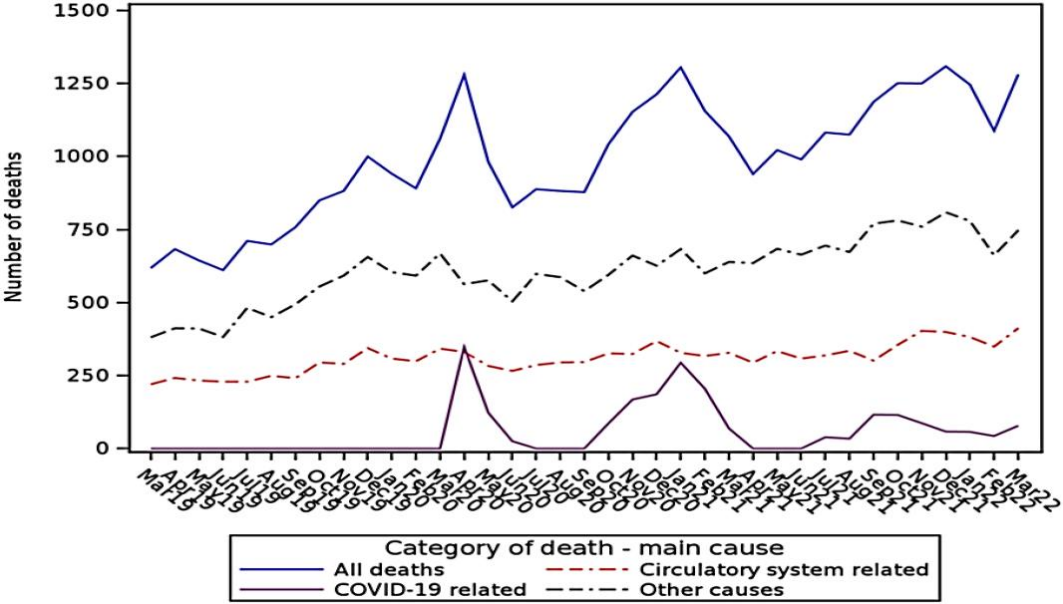
Numbers of deaths between march 2019 and march 2022, by main cause.

Overall, 8% of the cohort died during the 3-year follow-up period (*Figure 5*). The number of deaths per month rose throughout the follow-up period with peaks relating to COVID-19-related death peaks on top of expected seasonal variation. Of the deaths, 2573 (7%) were COVID-19 related and 2299 (6%) had COVID-19 attributed as the main cause. There was a high rate of COVID-19 vaccine uptake in this group, with 92% of the cohort having at least 1 vaccination (mean number of vaccinations 3, SD 0.5). The majority of those in the cohort who were unvaccinated did not have the opportunity, as 55% of those unvaccinated had died prior to the vaccine rollout (December 2020) and 65% by April 2021. As *Figure 5* shows, deaths with cardiovascular disease as the main cause did not vary substantially from month to month.

### Comparative cardiovascular outcomes

For the comparative portion of the analysis, patients who had used telemonitoring for less than 12 months were excluded, reducing the size of this group to 6123 individuals. Overall, 43095 (10%) of those eligible for this analysis experienced emergency hospital admission or death due to at least one of the composite cardiovascular events within up to 3 years after the index date. Of these, ACS was the most common (4.0% of the standard care group, 2.7% of the telemonitoring group) followed by HF (3.6% of the standard care group, 1.1% of the telemonitoring group), and stroke (2.2% of the standard care group, 1.4% of the telemonitoring group).

A matched analysis was performed to control for the differences in age, sex, ethnicity, SIMD, diabetes, number of classes of anti-hypertensive drugs, and index date before or after the onset of the COVID-19 pandemic, shown between the telemonitoring and standard care groups in *Table 1*. As not all patients could be matched, this analysis utilized a subgroup of the main cohort. comprising 192 529 patients, including 187 232 unique controls and 5297 unique telemonitoring patients. There were 3212 matched sets included in the matched analysis. There was a median of one telemonitoring patient per matched set (IQR 1–2, range 1–12), and a median of 15 control patients per matched set (IQR5–96, range 1–367). The conditional logistic regression analysis models were all based on *n* = 192,529, except the stratified and sensitivity analyses, which were based on a subset of this data. The results indicated that in these matched groups those who were using BP telemonitoring were less likely to be admitted to hospital with or die from ACS, stroke or uncontrolled heart failure compared with those who were not using BP telemonitoring [adjusted OR 0.665 (0.501–0.884), *P* = 0.0049], or to die through any cause [adjusted OR 0.602 (0.392–0.925), *P* = 0.0207] or be admitted to hospital for any cause [adjusted OR (0.776–0.922), *P* = 0.001]. *Table 2* shows the analysis stratified by index date (pre and post the start of the COVID-19 pandemic) with both the primary outcome and an extended outcome, which also included admissions for atrial fibrillation and cardiac revascularization. Although using the extended outcome increased numbers, it did not add additional discrimination. In addition, given the finding that 90% of the cohort were diagnosed pre March 2019 and the length of treatment was unknown, we also carried out a sensitivity analysis, which only included those who started antihypertensive treatment after the start of the study. This showed very similar odds ratios to the whole cohort analysis and a significant difference between the telemonitoring and standard care groups at the *P* < 0.05 level. This is also included in *Table 2*.

**Table 2:** Matched analysis results

	Overall adjusted odds ratio (95% CI) (Tele. *n* = 5297) (Control *n* = 187232) (Matched sets = 3212)	Index date before March 2020: adjusted odds ratio (95% CI) (Tele. *n* = 2540) (Control *n* = 178200) (Matched sets = 1701)	Index date March 2020 or later: adjusted odds ratio (95% CI) (Tele. *n* = 2757) (Control *n* = 9032) (Matched sets = 1511)	Sensitivity analysis only including patients who started treatment during the study Adjusted odds ratio (95% CI) (Tele. *n* = 1057) (Control *n* = 19702) (Matched sets = 2895)
**Composite cardiovascular outcome**	0.665 (0.501–0.884), *P* = 0.0049	0.932 (0.636–1.365), *P* = 0.7158	0.498 (0.336–0.739), *P* = 0.0005	0.440 (0.198–0.975), *P* = 0.0432
**Any death**	0.602 (0.392–0.925), *P* = 0.0207	0.788 (0.433–1.435), *P* = 0.4366	0.484 (0.268–0.875), *P* = 0.0162	0.230 (0.055–0.963), *P* = 0.0442
**Any hospital admission**	0.846 (0.776–0.922), *P* = 0.001	0.987 (0.879–1.107), *P* = 0.8199	0.713 (0.629–0.809), *P* < 0.001	0.731 (0.599–0.893), *P* = 0.0021
**Composite CV morbidity, including atrial fibrillation and coronary revascularization**	0.673 (0.542–0.837), *P* = 0.0004	0.812 (0.601–1.095), *P* = 0.1725	0.568 (0.420–0.770), *P* = 0.0003	0.430 (0.228–0.811), *P* = 0.0091

### Coverage of the cohort

The number of patients who were in the cohort initially (1 March 2019) was 99% of the estimate based on the Scottish Health Survey 2019.^12^ Analysis of data from GP records of 48740 people with a recorded diagnosis of hypertension from the sub-sample of 38 practices showed that the search used in this study would have identified 47069 (97%) of them. Thirty of these practices had some patients using the telemonitoring system, but none of the 1671 patients who did not have first-line anti-hypertensives as part of their treatment were doing so.

### Discussion

The key contribution of this study is that it demonstrated that patients using telemonitoring for routine long-term BP monitoring were less likely to suffer major cardiovascular events (ACS, stroke, or uncontrolled HF) than matched patients who were not using the service (adjusted OR 0.625, 95% CI 0.468–0.834, *P* = 0.001). The study showed that when people started using BP telemonitoring, their mean systolic BP reduced by 7.6 mmHg, similar to that seen in previous studies, and the reduction in major cardiovascular events would therefore be expected.^6,7^ Improved BP control tended to be achieved in the first 3 months of using the service and was maintained over 12 months. Margolis *et al*.^22^ found that a one year intervention using telemonitoring resulted in improved BP control which was maintained for a further year when the service was stopped, but then deteriorated. The BP telemonitoring service used in this study is provided for long-term use but further follow-up is needed to determine if the improved BP control is maintained over a number of years with continuous service use.

Use of the BP telemonitoring service grew particularly during the COVID-19 pandemic as it enabled remote monitoring. However, this was also a period of service disruption and there is some evidence that a proportion of non-telemonitoring users did not have their BP measured during that time.^17^ To compensate, a conservative approach to patient matching was adopted in this study, with new BP telemonitoring users being matched to people who had been diagnosed with hypertension in the same time period and would therefore have had an assessment of their BP management. Further, longer-term follow-up is required to compare the level and maintenance of BP control between telemonitoring users and non-users.

Compared with the whole group, telemonitoring users were younger, less likely to have diabetes, required fewer anti-hypertensive medications, and were less socio-economically deprived, all factors which placed them at lower risk. No significant difference was found in terms of ethnicity or sex, which is noteworthy given the very large sample size. It should be noted that the overall population in this study was not ethnically diverse, which is concordant with the Scottish Census figures for older age groups.^23^ Demographic differences between telemonitoring users and non-users found elsewhere have also included ethnicity.^24^ There is evidence that in a UK population, ethnicity influences cardiovascular risk in men, although possibly less so in women.^25^ Although this analysis showed that telemonitoring users had a lower risk of a cardiovascular event than matched non-users, there is always a possibility that there are other factors that influence the uptake of BP telemonitoring that are not available within these data. Given the outcome of this study, there is scope to explore whether it would be beneficial to extend BP telemonitoring to a wider demographic and higher-risk group of people with hypertension.

Overall, the impact of the COVID-19 pandemic on this group, in terms of including peaks in mortality and drops in hospital attendance, mirrored that of the Scottish population in general. Although hospital attendance patterns were changed by the COVID-19 pandemic, there was no evidence that the outcome measure used in this study (emergency admission for ACS, stroke, or uncontrolled HF) was affected. There was a high uptake of COVID-19 vaccination across the population, which is concordant with qualitative findings that this population saw themselves at high risk of severe COVID-19.^17^ However, the number of new diagnoses of hypertension halved during the first year of the pandemic, an issue also identified elsewhere,^15,16^ sparking concern that the number of people with undiagnosed hypertension in the population may be increasing, particularly as one study suggested there was a significant increase in new-onset hypertension during the pandemic.^26^ The potential increase in the number of people with undiagnosed hypertension suggests the need for increased attention to hypertension screening.

The strengths of this study are that the large numbers gave the study substantial power to identify differences in cardiovascular outcome and access to the telemonitoring data facilitated observation of the assumed causative mechanism, which was a reduction in BP. The limitation was that there was no access to BP data for those who were not telemonitoring and it was not clear how much BP monitoring for this group took place during the COVID-19 pandemic. The difference in cardiovascular outcomes cannot be generalized fully without longer term follow up during a more normal health service function in which surgery-measured BPs are available. Although a matched cohort analysis was used to control for differences between the telemonitoring and standard care groups, we cannot exclude confounding from underlying factors that were not considered in the matching procedure (i.e. residual confounding).^27^ Given that the results show that telemonitoring users were a lower risk group based on the characteristics available, it is also possible that other cardiovascular risk factors are not randomly distributed between the groups. Further work is required to investigate the impact of additional risk factors and cardiovascular outcomes at different levels of risk.

A further limitation to the study was the sampling strategy based on prescribing, which would exclude those people whose hypertension was being managed by medications other than first-line hypertensives and may include small numbers who were taking first-line anti-hypertensive medication for other indications. However, the GP practice-based sub-study indicated that the sampling strategy would have identified 97% of those who had a diagnosis of hypertension. The study also did not consider comparative health resource use by people who use telemonitoring and those who do not. Although avoiding a major cardiovascular event is likely to reduce resource use and there is recent evidence from other countries that BP telemonitoring is cost effective,^28^ there is a need for this to be explored further in a UK context.

In conclusion, the Connect Me BP telemonitoring service allowed patients to continue with BP monitoring when other monitoring services were disrupted due to the COVID-19 pandemic and there was a large increase in uptake of the service. Users had similar improved BP control to that seen in other studies and better cardiovascular outcomes than a matched control group of non-users. Further work is needed to explore cardiovascular outcomes outside the pandemic context.

## Supplementary Material

ztag069_Supplementary_Data

## Data Availability

All data used in this study was sourced from clinical and administrative records, linked, pseudonymized and made available to the authors on a secure server by the Electronic Data Research and Innovation Service (eDRIS), the Public Health Scotland (PHS) contact point for organizations or people wishing to make use of administrative datasets for research purposes. The authors cannot grant direct access to these datasets, but the approved, detailed data specifications required to recreate the datasets are provided as supplementary files.

## Lead author biography

Janet Hanley is an Associate Professor in the Cardiovascular Health Research Centre at Edinburgh Napier University. She is a graduate of the University of Edinburgh with a PhD from Queen Margaret University with a clinical background in community nursing, community service development, and NHS research management. Research interests include new technologies in health care. and has clinical trials of telemonitoring in a range of long-term conditions. Janet was involved in the successful development of a national blood pressure telemonitoring service in Scotland and is currently using clinical records to measure the impact on the incidence of major cardiovascular events.
