# Supplementary material for: Blood pressure telemonitoring and the incidence of cardiovascular events: a records based, matched patient analysis
Source: Eur Heart J Digit Health. 2026 May 26;7(5):ztag069. doi: 10.1093/ehjdh/ztag069 (PMC13208916; doi:10.1093/ehjdh/ztag069)
Supplement: ztag069_Supplementary_Data [file ztag069_supplementary_data.zip › supplementary material.docx]

# Supplementary Material

## Connect Me BP

Connect Me BP (27) (formerly known as Scale-Up BP which used the Florence telehealth system (28) with additional practice reporting mechanisms), involves patients checking their BP at predefined frequencies determined by their healthcare team, using a British Hypertension Society approved electronic sphygmomanometer. The patient is shown how to use the BP machine and to record results on a third-party website. An action plan is agreed, based on a standard template although thresholds and actions can be agreed to suit the individual. Automated reminders are sent to participating people via SMS, email or landline to check their BP. Patients using SMS or email are sent an automated message on receipt of their BP reading, confirming if the reading is within range or should be repeated if high. An essential element of the intervention is the understanding patients are expected to take an active role in their care and actions on high readings are the responsibility of the patient. BP readings are summarised into weekly, monthly, three or six-monthly reports with averages already calculated, the frequency determined by the frequency of BP readings requested in the protocol the patient is following and sent to the GP practice for clinician review. The level of patient follow up is determined by whether BP readings signal controlled or uncontrolled hypertension. Any changes required after clinician review are communicated to the patient by telephone, email, letter, or text at the clinician’s discretion, with patients only invited to attend the surgery if blood tests or physical examination are required.

## Drug list used to select the cohort

Amlodipine (0206020A0)

Diltiazem hydrochloride (0206020C0)

Felodipine (0206020F0)

Isradipine (0206020I0)

Lacidipine (0206020K0)

Lercanidipine hydrochloride (0206020L0)

Nicardipine hydrochloride (0206020Q0)

Nifedipine (0206020R0)

Nimodipine (0206020M0)

Nisoldipine (0206020W0)

Trimetazidine hydrochloride (0206020B0)

Valsartan/amlodipine (0206020Z0)

Verapamil hydrochloride (0206020T0)

Aliskiren (0205053A0)

Azilsartan medoxomil (0205052AD)

Candesartan cilexetil (0205052C0)

Captopril (0205051F0)

Cilazapril (0205051E0)

Co-zidocapt (Hydrochlorothiazide/captopril) (0205051G0)

Enalapril maleate (0205051I0)

Enalapril maleate with diuretic (0205051H0)

Eprosartan (0205052W0)

Fosinopril sodium (0205051J0)

Imidapril hydrochloride (0205051W0)

Irbesartan (0205052I0)

Irbesartan with diuretic (0205052A0)

Lisinopril (0205051L0)

Lisinopril with diuretic (0205051K0)

Losartan potassium (0205052N0)

Losartan potassium with diuretic (0205052P0)

Moexipril hydrochloride (0205051C0)

Olmesartan medoxomil (0205052B0)

Olmesartan medoxomil/amlodipine (0205052AB)

Olmesartan medoxomil/amlodipine/hydrochlorothiazide (0205052AC)

Olmesartan medoxomil/hydrochlorothiazide (0205052Y0)

Perindopril arginine (0205051Y0)

Perindopril arginine with diuretic (0205051Z0)

Perindopril erbumine (0205051M0)

Perindopril erbumine with diuretic (0205051N0)

Perindopril tosilate (0205051AA)

Perindopril tosilate/indapamide (0205051AB)

Perindopril with calcium channel blocker (0205051AC)

Quinapril hydrochloride (0205051Q0)

Quinapril hydrochloride with diuretic (0205051P0)

Ramipril (0205051R0)

Ramipril with calcium channel blocker (0205051S0)

Sacubitril/valsartan (0205052AE)

Telmisartan (0205052Q0)

Telmisartan with diuretic (0205052R0)

Trandolapril (0205051U0)

Trandolapril with calcium channel blocker (0205051V0)

Valsartan (0205052V0)

Valsartan with diuretic (0205052X0)

## ICD-10 coding for primary outcome

| **Composite CV outcome**  Stroke | **ICD10 codes**  I61, I63, I64 |
| --- | --- |
| TIA (Transient cerebral ischaemic attack) | G45 |
| MI (Myocardial infarction) | I21, I22 |
| ACS (Acute Coronary Syndrome) | I200 and I24 |
| Uncompensated cardiac failure | I500, I509, I110, I130, I132, I50 |

## Table showing change in BP by month after start of telemonitoring

| months from first reading | Mean Systolic BP | Number of patients submitting readings during the month* | Std. Deviation |
| --- | --- | --- | --- |
| 0 | 140.5 | 1569 | 14.6 |
| 1 | 136.0 | 924 | 13.3 |
| 2 | 134.0 | 812 | 12.7 |
| 3 | 132.9 | 684 | 12.0 |
| 4 | 132.8 | 615 | 12.1 |
| 5 | 132.4 | 575 | 12.0 |
| 6 | 132.4 | 536 | 12.5 |
| 7 | 132.6 | 495 | 11.5 |
| 8 | 132.7 | 485 | 12.2 |
| 9 | 132.3 | 446 | 11.7 |
| 10 | 131.7 | 417 | 11.5 |
| 11 | 131.6 | 395 | 11.3 |
| 12 | 130.8 | 362 | 11.3 |
| 13 | 131.1 | 333 | 10.2 |
| 14 | 131.0 | 293 | 11.6 |

*The frequency at which people were asked to submit readings was determined by the clinician and was also subject to change

Table comparing characteristics of people in matched dataset

**Comparing baseline characteristics of matched dataset**

|  | **Control (n=187232)** | **Telemonitoring (n=5297)** | **Overall (n=192529)** |
| --- | --- | --- | --- |
| Time period  Pre-March 2020  Post-March 2020 | 178200  9032 | 2540  2757 | 180740  11789 |
| Number of anti-hypertensive medications  1  2  3  4 | 91716  76006  17605  1905 | 2719  1835  604  139 | 94435  77841  18209  2044 |
| Ethnicity cohort  White  Other/Missing | 106343  80889 | 2825  2472 | 109168  83361 |
| Diabetes  No  Yes | 173568  13664 | 4684  613 | 178252  14277 |
| SIMD category  1  2  3  4  5 | 18286  43168  35160  35764  54854 | 636  1216  955  1010  1480 | 18922  44384  36115  36774  56334 |
| Sex  Male  Female | 101599  85633 | 2802  2495 | 104401  88128 |
| Age  n  mean  sd  median  1^st^ Quartile  3^rd^ Quartile | 187232  63.79  9.46  64  57  71 | 5297  59.49  10.69  60  52  67 | 192529  63.67  9.52  64  57  71 |

## Data Specification

This will be uploaded as excel files
